# Short-Term Effects of Hyperthermic Intraperitoneal Chemotherapy on Patients Following Cytoreductive Surgery: Retrospective Analysis From a Tertiary Care Center in Saudi Arabia

**DOI:** 10.7759/cureus.102860

**Published:** 2026-02-02

**Authors:** Salman Alahmad, Sohail Alelaiyan, Hadeel Helmi, Faiza Alrozaini, Wed Alwabel, Ghala Albaqami, Aimn W Abdujawad, Nayef Alzahrani

**Affiliations:** 1 General Surgery, King Abdulaziz Medical City, Riyadh, SAU; 2 College of Medicine, King Saud Bin Abdulaziz University for Health Sciences, Riyadh, SAU; 3 National Guard Health Affairs, King Abdulaziz Medical City, Riyadh, SAU

**Keywords:** cytoreductive surgery, hipec, morbidity, peritoneal cancer index, peritoneal surface malignancy

## Abstract

Introduction

Cytoreductive surgery (CRS) with the use of hyperthermic intraperitoneal chemotherapy (HIPEC) is the standard of treatment for peritoneal surface malignancy. Increasing evidence demonstrates the survival benefit of CRS combined with HIPEC, with the completion of cytoreduction being a key factor in prognosis. This study aimed to evaluate the impact of HIPEC on short-term postoperative outcomes in patients undergoing CRS and to assess factors associated with postoperative morbidity.

Methods

This retrospective cohort study was conducted using a maintained database of patients with peritoneal surface malignancies who underwent CRS with and without HIPEC at a tertiary care center in Riyadh, Saudi Arabia, from January 1, 2021, to November 1, 2024.

Results

A total of 95 patients were included, 52 of whom underwent CRS alone and 43 received CRS combined with HIPEC. Demographic and clinical characteristics were largely similar between groups; however, patients who underwent CRS and HIPEC had higher preoperative albumin levels (p = 0.007) and more frequent previous surgeries (p = 0.020). Operative time was significantly longer in the CRS and HIPEC group (p < 0.001), and stoma creation was more common (p = 0.014). Overall, postoperative complications were fewer in the CRS and HIPEC group. The 30-day mortality rate was 0% for both groups, and the Peritoneal Cancer Index (PCI) was the only predictor of severe complications (p = 0.047).

Conclusion

Complete cytoreduction remains the standard in the treatment of peritoneal surface malignancies to maximize survival potential. The addition of HIPEC does not appear to increase short-term postoperative outcomes, while disease burden, as reflected by the PCI, remains the primary independent determinant of postoperative risk.

## Introduction

Hyperthermic intraperitoneal chemotherapy (HIPEC) is an advanced oncologic intervention that combines surgical cytoreduction with the direct administration of heated chemotherapeutic agents into the peritoneal cavity. This technique targets peritoneal metastasis, which is commonly associated with malignancies such as colorectal, ovarian, and gastric cancer.

In Saudi Arabia, the adoption of HIPEC has been gaining traction, propelled by the rising incidence of cancer and the need for innovative treatment options. According to the Saudi Cancer Registry, colorectal cancer is the second most common cancer among Saudi males and the third among females, with a significant annual increase in cases. Ovarian cancer also presents a growing concern, necessitating advanced therapeutic approaches like HIPEC [1].

Saudi Arabia has been investing heavily in healthcare infrastructure and medical education, enabling the integration of advanced oncologic procedures like HIPEC across multiple centers in the Kingdom. Leading medical institutions in Riyadh have adopted HIPEC as part of their surgical oncology practice. Additionally, HIPEC offers several advantages over systemic chemotherapy, including localized high-dose drug delivery, reduced systemic toxicity, and enhanced cytotoxic efficacy due to hyperthermia [2]. The hyperthermic component, typically maintained between 41°C and 43°C, increases cellular membrane permeability, thereby facilitating higher intracellular concentrations of chemotherapeutic agents and enhancing cytotoxicity against malignant cells [3].

Cytoreductive surgery with or without HIPEC (CRS ± HIPEC) is now a standard treatment for peritoneal surface malignancies [4]. These extensive procedures can cause significant tissue damage and subsequent inflammation, with reported major complication rates reaching up to 60% [5], necessitating the need to integrate protocols such as “enhanced recovery after surgery” (ERAS) and “enhanced recovery after anesthesia” (ERAA). Postoperative recovery and quality-of-life outcomes following CRS-HIPEC have been variably reported, with an initial physical and psychological burden followed by potential improvement in functional and social domains in many patients [6,7]. In one study, the mortality of 30-day in-hospital following CRS and HIPEC was 2.5%, with mortality associated primarily with the extent and complexity of cytoreductive surgery [8].

Despite its promise, the widespread use of HIPEC in Saudi Arabia faces challenges, including the need for specialized surgical expertise and a high economic burden [9]. This study aimed to evaluate the impact of HIPEC on short-term postoperative outcomes in patients undergoing CRS and to assess the factors associated with postoperative morbidity.

## Materials and methods

Study design

This study was conducted at King Abdulaziz Medical City, National Guard Health Affairs, Riyadh, Saudi Arabia, which is a publicly funded tertiary care center. Data were collected and analyzed from a retrospectively maintained database of peritoneal surface malignancies. All patients who underwent CRS, with and without the use of HIPEC, from January 1, 2021, to November 1, 2024, were included. Institutional review board approval was obtained on August 13, 2025, from the Institutional Review Board of King Abdullah International Medical Research Center, with approval number 0000035324. The study was designed to evaluate short-term postoperative outcomes following CRS with and without HIPEC, with additional analysis of factors associated with postoperative morbidity.

Participants

Patient demographics, primary tumor characteristics, intraoperative findings, and short-term surgical outcomes and complications were recorded. Inclusion criteria encompassed all patients undergoing CRS, with or without HIPEC, who were discussed at the multidisciplinary team meeting, informed about the procedures, and deemed eligible for complete cytoreduction. Exclusion criteria included patients younger than 14 years and those undergoing emergency surgery.

Technique

CRS was performed by the same surgical team using a standardized and consistent operative approach. The main goal of the surgery was maximal cytoreduction, with resection of all identifiable peritoneal disease when feasible. The extent of resection and combined organ resection was assessed and determined intraoperatively based on disease burden.

Hyperthermic intraperitoneal chemotherapy (HIPEC) was generally considered for patients with peritoneal disease and carcinomatosis or pseudomyxoma peritonei, particularly those with a low disease burden with PCI less than 15 and near complete cytoreduction reaching CC-1.

Following the completion of cytoreductive surgery, intraperitoneal chemotherapy was administered in indicated patients using the open “coliseum” technique (O-HIPEC), as described by Sugarbaker [10]. After placement of inflow and outflow catheters, the abdominal wall was suspended using retractors to create a temporary perfusion field (“coliseum”), allowing continuous manual agitation to ensure uniform distribution of the perfusate. Chemotherapy was administered under hyperthermic conditions, with target inflow temperatures of approximately 43°C and intraperitoneal temperatures maintained between 41°C and 42°C. The duration of HIPEC perfusion typically ranged from 60 to 90 minutes, depending on the chemotherapeutic agent and protocol. Chemotherapy agents were selected according to primary tumor type.

Statistical analysis

Chi-square test was applied to assess associations between categorical variables across groups. Frequencies and percentages were reported alongside p-values to determine statistical significance. The independent samples t-test was used to compare continuous variables. Mean values, standard deviations, and corresponding p-values were reported to identify significant differences. Binary logistic regression was performed to identify factors associated with the severity of complications (a Clavien-Dindo (CD) score of 3a or more was considered as severe complications). Odds ratios (OR) with 95% confidence intervals (CI) and p-values were calculated to evaluate the strength and significance of associations. The results were presented with appropriate statistical significance thresholds (p < 0.05), highlighting significant predictors and differences between groups.

## Results

All patients who underwent CRS during the study period were included, except those meeting the exclusion criteria. The patients were divided into two groups: those who underwent CRS alone (52 patients) and those who received CRS combined with HIPEC (43 patients). All patients underwent complete cytoreduction.

Table 1 shows the demographics and characteristics of the patients (average age: 56.80 years, SD = 13.46, for the CRS group; 52.90 years, SD = 15.22, for the CRS and HIPEC group; p = 0.188). Gender distribution was similar, with 23 (44.2%) males and 29 (55.8%) females in the CRS group, and 21 (48.8%) males and 22 (51.2%) females in the CRS and HIPEC group (p = 0.654). Body mass index (BMI) averaged 25.16 (SD = 5.66) in the CRS group, and 24.09 (SD = 5.39) in the CRS and HIPEC group (p = 0.350). The American Society of Anesthesiologists (ASA) Physical Status classification showed no significant differences between the groups (p = 0.790). Smoking status was similar across both groups, with the majority being non-smokers (p = 0.846). Comorbidities, including cardiac and respiratory conditions and endocrine disorders, were relatively evenly distributed between groups, with no statistically significant differences observed. 

**Table 1:** Patient demographic and other characteristics. *p-values < 0.05 are considered statistically significant.

	CRS alone (n = 52)	CRS and HIPEC (n = 43)	p-value
Age at surgery (years, mean ± SD)	56.80 ± 13.46	52.90 ± 15.22	0.188
Gender, n (%)			0.654
Male	23 (44.2%)	21 (48.8%)	-
Female	29 (55.8%)	22 (51.2%)	-
Weight (kg, mean ± SD)	66.88 ± 18.18	62.94 ± 14.10	0.248
BMI (mean ± SD)	25.16 ± 5.66	24.09 ± 5.39	0.350
ASA, n (%)			0.790
2	18 (34.6%)	14 (32.5%)	-
3	32 (61.6%)	26 (60.5%)	-
4	2 (3.8%)	3 (7.0%)	-
Smoking, n (%)			0.846
No	50 (96.2%)	41 (95.3%)	-
Yes	2 (3.8%)	2 (4.7%)	-
Comorbidities			
Cardiac, n (%)			0.341
No	34 (65.4%)	32 (74.4%)	-
Yes	18 (34.6%)	11 (25.6%)	-
Resp, n (%)			0.258
No	43 (82.7%)	39 (90.7%)	-
Yes	9 (17.3%)	4 (9.3%)	-
Endocrine DM, n (%)			0.097
No	33 (63.5%)	34 (79.1%)	-
Yes	19 (36.5%)	9 (20.9%)	-
Endocrine thyroid, n (%)			0.202
No	44 (84.6%)	40 (93.0%)	-
Yes	8 (15.4%)	3 (7.0%)	-
Renal, n (%)			0.194
No	50 (96.2%)	43 (100.0%)	-
Yes	2 (3.8%)	0 (0.0%)	-

As provided in Table 2, the primary tumor origin was predominantly colorectal in both groups (31 (59.6%) in CRS and 26 (60.5%) in CRS and HIPEC; p = 0.933). Notably, ovarian primary tumors were present in five (9.6%) from the CRS group and none in the CRS and HIPEC group (p = 0.037). The mean hemoglobin levels were nearly identical between the two groups (111.98 ± 20.15 g/L in the CRS group; 112.30 ± 18.74 g/L in the CRS and HIPEC group). This difference was statistically significant (p = 0.012), indicating a slightly higher hemoglobin level in the CRS and HIPEC group. Preoperative laboratory values highlighted a significant difference in albumin levels, with higher values in the CRS and HIPEC group (37.81 ± 5.67 g/L vs. 34.54 ± 5.76 g/L; p = 0.007). Previous surgeries for primary tumors were more frequent in the CRS and HIPEC group (26 (60.5%) vs. 19 (36.5%); p = 0.020). The choice of HIPEC drug varied among CRS and HIPEC patients. Mitomycin C was the predominant agent, used in 36 (85.7%) patients. Stoma creation was significantly more common in the CRS and HIPEC group (15 (34.9%) vs. 7 (13.5%); p = 0.014).

**Table 2:** Disease-related variables and intraoperative findings. *p-values < 0.05 are considered statistically significant. PCI: Peritoneal Cancer Index, HIPEC: hyperthermic intraperitoneal chemotherapy, CRS: cytoreductive surgery.

Variables/findings	CRS (n = 52)	CRS and HIPEC (n = 43)	p-value
Primary tumor			
Colorectal, n (%)			0.933
Yes	31 (59.6%)	26 (60.5%)	-
Appendix, n (%)			0.211
Yes	6 (11.5%)	9 (20.9%)	-
Gastric, n (%)			0.269
Yes	0 (0%)	1 (2.3%)	-
Small bowel, n (%)			0.405
Yes	3 (5.8%)	1 (2.3%)	-
Ovarian, n (%)			0.037*
Yes	5 (9.6%)	0 (0%)	-
Mesothelioma, n (%)			0.892
Yes	1 (1.9%)	1 (2.3%)	-
Sarcoma, n (%)			0.779
Yes	4 (7.7%)	4 (9.3%)	-
Gallbladder, n (%)			0.892
Yes	1 (1.9%)	1 (2.3%)	-
Pre-op labs			
Hgb (gm/L, mean ± SD)	111.98 ± 20.15	112.30 ± 18.74	0.012*
Albumin (g/L, mean ± SD)	34.54 ± 5.76	37.81 ± 5.67	0.007*
Creatinine (umol/L mean ± SD)	64.94 ± 17.90	61.58 ± 13.64	0.314
CA 19-9 (U/mL, mean ± SD)	144.53 ± 218.95	120.21 ± 329.77	0.728
CA 125 (U/mL, mean ± SD)	67.11 ± 109.02	29.32 ± 37.42	0.106
CEA level (ng/mL, mean ± SD)	77.14 ± 235.73	76.79 ± 287.84	0.995
Previous surgery for primary tumor, n (%)			0.020*
Yes	19 (36.5%)	26 (60.5%)	-
Redo CRS, n (%)			0.724
Yes	6 (11.5%)	4 (9.3%)	-
PCI (mean ± SD)	11.34 ± 9.99	13.61 ± 10.72	0.355
HIPEC drug: peritoneal, n (%)			<0.001*
Cisplatin	0 (0.0%)	3 (7.1%)	-
Cisplatin and doxorubicin	0 (0.0%)	2 (4.8%)	-
Cisplatin and mitomycin C	0 (0.0%)	1 (2.4%)	-
Mitomycin C	0 (0.0%)	36 (85.7%)	-
Extent of CRS			
Diaphragmatic stripping, n (%)			0.215
Yes	9 (17.3%)	12 (27.9%)	-
Stoma creation, n (%)			0.014*
Yes	7 (13.5%)	15 (34.9%)	-
Omentectomy, n (%)			0.372
Yes	33 (63.5%)	31 (72.1%)	-
Gastric resection, n (%)			0.808
Yes	3 (5.8%)	2 (4.7%)	-
Pancreatectomy, n (%)			0.544
Yes	4 (7.7%)	2 (4.7%)	-
Liver resection, n (%)			0.544
Yes	6 (11.5%)	9 (20.9%)	-
Cholecystectomy, n (%)			0.764
Yes	33 (63.5%)	26 (60.5%)	-
Splenectomy, n (%)			0.062
Yes	4 (7.7%)	9 (20.9%)	-
Small bowel resection, n (%)			0.785
Yes	22 (42.3%)	17 (39.5%)	-
Colon resection, n (%)			0.169
Yes	35 (67.3%)	23 (53.5%)	-
Appendectomy, n (%)			0.569
Yes	13 (25.0%)	13 (30.2%)	-
Rectal resection, n (%)			0.676
Yes	8 (15.4%)	8 (18.6%)	-
Ureter resection, n (%)			0.361
Yes	1 (1.9%)	0 (0.0%)	-
Bladder resection, n (%)			0.450
Yes	6 (11.5%)	3 (7%)	-
Nephrectomy, n (%)			0.194
Yes	2 (3.8%)	0 (0.0%)	-
Hysterectomy, n (%)			0.333
Yes	7 (13.5%)	9 (20.9%)	-
Salpingo-oophorectomy, n (%)			0.900
Yes	20 (38.5%)	16 (37.2%)	-
Orchidectomy, n (%)			0.361
Yes	1 (1.9%)	0 (0%)	-
Abdominal wall resection, n (%)			0.989
Yes	6 (11.5%)	5 (11.6%)	-

The mean operative time was significantly longer in the CRS and HIPEC group (499.44 ± 108.60 minutes) compared to the CRS group (332.27 ± 112.77 minutes; p < 0.001) (Table 3). The rate of red blood cell transfusion was 26 (50%) in the CRS group, and 19 (44.2%) in the CRS and HIPEC group, with no statistically significant difference between the groups (p = 0.572). No significant differences were observed in postoperative ICU admission rates, length of ICU stay, or hospital stay duration. It is important to note that the mortality rate at the 30-day mark is 0 (0%) for both groups.

**Table 3:** Short-term surgical outcomes. *p-values < 0.05 are considered statistically significant. PRBC: packed red blood cells, ICU: intensive care unit, HDU: high dependency unit.

Short-term surgical outcomes	CRS (n = 52)	CRS and HIPEC (n = 43)	p-value
Operative time (min, mean ± SD)	332.27 ± 112.77	499.44 ± 108.60	<0.001*
PRBC transfusion, n (%)			0.572
Yes	26 (50.0%)	19 (44.2%)	-
First bowel motion post-op (days, mean ± SD)	5.92 ± 4.27	5.23 ± 2.37	0.346
ICU/HDU admission, n (%)			0.357
Yes	47 (90.4%)	41 (95.3%)	-
ICU re-admission, n (%)			0.194
Yes	2 (3.8%)	0 (0.0%)	-
Length of ICU stay (days, mean ± SD)	4.98 ± 7.44	3.35 ± 1.80	0.164
Length of postoperative stay (days, mean ± SD)	17.62 ± 13.13	15.40 ± 6.90	0.320
Total hospital stay (days, mean ± SD)	21.08 ± 14.00	18.81 ± 6.96	0.337
ER visit within 30 days, n (%)			0.883
Yes	15 (28.8%)	13 (30.2%)	-
Re-admission within 30 days, n (%)			0.511
Yes	5 (9.6%)	6 (14.0%)	-
Mortality at 30-day mark, n (%)			-
Yes	0 (0.0%)	0 (0.0%)	-

Table 4 presents a comparison of postoperative complications between patients who underwent CRS alone (n = 52) and those who underwent CRS with HIPEC (n = 43). Overall, the CRS and HIPEC group experienced fewer complications, with an average of 0.58 ± 0.73 complications per person compared to 1.33 ± 1.37 in the CRS-only group (p = 0.002). A statistically significantly lower incidence of symptomatic pleural effusion was observed in the CRS and HIPEC group (2, 4.7%) compared to the CRS group (16, 30.8%) (p = 0.001). Additionally, urinary retention was less frequent in the CRS and HIPEC group (1, 2.3%) than in the CRS group (9, 17.3%) (p = 0.018). Although other complications such as pulmonary embolism, pneumothorax, venous thrombosis, abdominal collection, ileus, bowel obstruction, urinary tract infection (UTI), cerebral vascular lesion, and pancreatic leak were less frequent in the CRS and HIPEC group, the differences did not reach statistical significance (p > 0.05 for each).

**Table 4:** Complications. *p-values < 0.05 are considered statistically significant.

Complication	CRS (n = 52)	CRS and HIPEC (n = 43)	p-value
Pulmonary embolism, n (%)			0.131
Yes	9 (17.3%)	3 (7.0%)	-
Pneumothorax, n (%)			0.544
Yes	4 (7.7%)	2 (4.7%)	-
Venous thrombosis, n (%)			0.144
Yes	7 (13.5%)	2 (4.7%)	-
Abdominal collection, n (%)			0.724
Yes	6 (11.5%)	4 (9.3%)	-
Intra-abdominal bleeding, n (%)			0.779
Yes	4 (7.7%)	4 (9.3%)	-
Ileus, n (%)			0.244
Yes	4 (7.7%)	1 (2.3%)	-
Bowel obstruction, n (%)			0.194
Yes	2 (3.8%)	0 (0.0%)	-
Fistula, n (%)			0.269
Yes	0 (0.0%)	1 (2.3%)	-
UTI, n (%)			0.361
Yes	1 (1.9%)	0 (0.0%)	-
Wound infection, n (%)			0.512
Yes	3 (5.8%)	4 (9.3%)	-
Cerebral vascular lesion, n (%)			0.109
Yes	3 (5.8%)	0 (0.0%)	-
Symptomatic pleural effusion, n (%)			0.001*
Yes	16(30.8%)	2(4.7%)	-
Pneumonia, n (%)			0.269
Yes	0 (0.0%)	1 (2.3%)	-
Pancreatic leak, n (%)			0.361
Yes	1 (1.9%)	0 (0.0%)	-
Urinary retention, n (%)			0.018*
Yes	9 (17.3%)	1 (2.3%)	-
CD score, n (%)			0.790
>3a	21 (55.3%)	21 (58.3%)	-
≤3a	17 (44.7%)	15 (41.7%)	-
Average complications/person	1.33 ± 1.37	0.58 ± 0.73	0.002*

Table 5 summarizes the association between various factors and the severity of the Clavien-Dindo (CD) score, expressed as ORs with corresponding 95% CIs, chi-square values, and p-values. A binary logistic regression was performed to measure if any variable would be a predictor for a higher CD score (≤3a). The Peritoneal Cancer Index (PCI) was the only factor significantly associated with the severity of complications, with an OR of 1.137 (95% CI: 1.002-1.290, p = 0.047), indicating that for every one-point increase in PCI, the odds of experiencing more severe complications (as measured by the CD score) increase by 13.7%. Other factors, including age at surgery (OR: 0.941, p = 0.068), gender (OR: 1.502, p = 0.607), BMI (OR: 1.012, p = 0.862), and use of HIPEC (OR: 0.324, p = 0.152), did not demonstrate statistically significant associations with the severity of complications. Similarly, ASA class showed a trend toward increased odds of severe complications in patients classified as ASA 3 (OR: 4.261, p = 0.119) and ASA 4 (OR: 13.059, p = 0.109), but these results were not statistically significant. Regarding primary tumor types, none of the tumor categories (colorectal, appendix, gastric, small bowel, ovarian, mesothelioma, sarcoma, and gallbladder) were significantly associated with the severity of the CD score (p > 0.999 for all). ORs for individual primary tumor subtypes were unstable due to small subgroup sizes and sparse event counts and should therefore be interpreted with caution while focusing on statistically stable predictors, particularly PCI. Additionally, chi-square values are reported where applicable.

**Table 5:** The association between patient, perioperative, and tumor factors and severity of Clavien-Dindo (CD) score. Odds ratios (OR) with 95% confidence intervals (CI) are presented, and chi-square (χ²) values are included where applicable. ORs for primary tumor subtypes were unstable due to small subgroup sizes and sparse event counts and should be interpreted with caution. *p-values < 0.05 are considered statistically significant.

Variable	χ² (Pearson)	B^a^	OR	95% CI lower	95% CI upper	p-value
Age at surgery	-	-0.061	0.941	-	-	0.068
Gender	0.274	0.407	1.502	-	-	0.607
BMI	0.032	0.012	1.012	-	-	0.862
Smoking	0.000	-21.805	0.000	-	-	1.000
ASA class						
ASA 2 (Ref)	-	-	1.000	-	-	-
ASA 3	2.435	1.450	4.261	-	-	0.119
ASA 4	2.575	2.570	13.059	-	-	0.109
Use of HIPEC	0.071	-1.128	0.324	-	-	0.152
Pre-op hemoglobin	-	0.000	1.000	-	-	0.990
Pre-op albumin	-	0.107	1.113	-	-	0.175
PCI	-	0.128	1.137	1.002	1.290	0.047*
pRBC transfusion	0.121	-0.304	0.738	-	-	0.726
Primary tumor						
Colorectal	0.003	22.383	52.60 × 10⁸	-	-	1.000
Appendix	0.002	22.148	41.57 × 10⁸	-	-	1.000
Gastric	0.001	-0.226	0.798	-	-	1.000
Small bowel	0.004	22.970	94.59 × 10⁸	-	-	1.000
Ovarian	0.003	42.570	30.76 × 10¹⁷	-	-	0.999
Mesothelioma	0.005	43.653	90.81 × 10¹⁷	-	-	0.999
Sarcoma	0.002	19.706	36.15 × 10⁷	-	-	1.000
Gallbladder	0.004	41.028	65.78 × 10¹⁶	-	-	0.999

## Discussion

In this study, we evaluated short-term postoperative outcomes in patients who underwent CRS with and without HIPEC. Our findings demonstrate that the addition of HIPEC did not increase the 30-day mortality nor the rate of severe postoperative complications. Although operative time was significantly longer in the CRS and HIPEC group, the overall postoperative morbidity did not differ from that in patients who received CRS alone. Notably, the PCI emerged as the only independent predictor of severe complications, whereas the use of HIPEC itself was not associated with increased postoperative risk. Since the description of complete peritonectomy in CRS by Paul H. Sugarbaker in 1995 [10], advancements have been made to standardize the role of CRS and the concomitant use of HIPEC in the treatment of peritoneal surface malignancies. A study conducted by Verwaal et al. showcased an increased survival in patients who underwent CRS and HIPEC compared to systemic therapy alone [11]. Although mortality remained minimal, morbidity rates were high in patients who underwent CRS and HIPEC. Similarly, another study demonstrated significant patient morbidity post-HIPEC, regardless of their successful outcomes. Complications were associated with the longer operating times, mainly pulmonary and renal, as well as surgical site infections and anastomotic leak [12]. In contrast, our findings did not demonstrate higher overall morbidity in patients undergoing CRS and HIPEC, despite longer operative times, suggesting that HIPEC can be incorporated without increasing short-term surgical risk in suitable candidates.

Furthermore, skepticism remains prevalent in the overall benefit and added value of HIPEC, independent of reaching a complete CRS (CC-0). The CC score has been found to be a strong prognostic factor in CRS with HIPEC across multiple studies, with CC-0 resection being linked to remarkably improved outcomes [13]. Likewise, the PCI is another recognized indicator for measuring the disease burden and predicting the likelihood of reaching a complete reduction (CC-0). A higher PCI score indicates extensive disease involvement and is greatly linked to poorer postoperative morbidity and long-term prognosis, in addition to decreasing the probability of achieving a total cytoreduction [12]. Comparably, PCI was the only independent predictor of severe postoperative complications in our analysis, highlighting its role as a key determinant of surgical outcome.

The recent PRODIGE 7 trial, published in 2021, with the aim to address and evaluate the specific role of HIPEC in combination with CRS in patients with colorectal cancer-related peritoneal surface malignancy, showed no statistically significant survival benefit in the group who received HIPEC, compared to those who underwent CRS alone. However, their findings were potentially influenced by the nature of their study design, which included the use of oxaliplatin-based HIPEC, short perfusion duration, and low PCI scores in selected patients. The authors also emphasized that the most significant prognosis predictor was the ability to achieve CC-0, further highlighting the importance of surgical clearance for a better outcome [14]. Similarly, a retrospective cohort study done by the Peritoneal Surface Oncology Group International (PSOGI) and BIG RENAPE collaborative groups matched 30 women with malignant peritoneal disease of endometrial cancer origin treated with CRS and HIPEC, and compared them to another 30 matched women with similar disease characteristics who only underwent CRS alone. The results revealed a median overall survival of 19.2 months in the HIPEC group versus 29.7 months in the CRS-only group, showing no survival advantage from receiving both CRS and HIPEC [15]. Again, these outcomes may have been influenced by differences in tumor development, timing of intervention, and patient factors, rather than representing a definitive disadvantage from HIPEC addition to treatment. The results of our study further support that HIPEC is unlikely to increase short-term morbidity, while illustrating the impact disease burden has on postoperative risk outcomes. Consequently, further prospective studies are needed to identify patient subgroups who may truly benefit from a combination HIPEC and CRS treatment strategy.

The PCI has been established as an effective guide to patient selection with specific values proposed to help identify the patients who are more likely to benefit from CRS, with or without the addition of HIPEC. This approach helps avoid selecting patients who will have minimal benefit, such as those with extensive disease. Notably, the subgroup analysis from the PRODIGE 7 trial showed longer median overall survival in patients with a PCI score of 11-15 who underwent combined CRS and HIPEC therapy [14], suggesting a significant benefit in intermediate risk groups and inviting future endeavors to solidify this finding. Overall, PCI is significantly associated with postoperative complications. However, this is most likely due to the extent of cytoreduction required in high PCI patient groups, who undergo a more aggressive surgery to successfully reduce the extensive disease.

Also, advanced age and comorbidities are major risk factors for postoperative morbidity. Patients over the age of 65 have shown to exhibit a significant increase in the likelihood of developing CD grade 3a complications, even in those without underlying conditions or comorbidities [16]. While age and comorbidities were not independently associated with severe complications in our study, this finding may be attributable to careful patient selection through multidisciplinary evaluation. In general, the predictors of postoperative morbidity identified in this study are consistent with those reported in the current literature. This study has several limitations. First, the retrospective design introduces the risk of selection bias, as treatment decisions were based on clinical judgement and institutional criteria; thus, patients were not randomized into treatment groups. Moreover, patient suitability for undergoing CRS and HIPEC was discussed in a weekly multidisciplinary team meeting, which may have led to excluding patients with poor performance status, significant comorbidity, or severe malnutrition. As a result, the study population may represent a more favorable cohort. In addition, due to the retrospective nature of the study, detailed technical parameters of HIPEC administration beyond the standardized technique description were not uniformly documented and could not be further analyzed. Additionally, the preoperative status of systemic chemotherapy data was incomplete, which limited the analysis of its impact on the outcomes. Despite these limitations, the findings of this study are similar to those reported in the current literature with regard to the short-term prognostic outcomes across various malignancies.

## Conclusions

CRS remains the cornerstone of management for peritoneal surface malignancies, with the completeness of cytoreduction (CC-0) serving as the most important prognostic determinant of survival. The addition of HIPEC does not appear to significantly increase postoperative morbidity, while disease burden, as depicted by PCI, remains an independent predictor of postoperative morbidity. While our findings align with the existing literature, further large-scale, prospective studies are warranted to better define the subgroups of patients who may derive the greatest survival advantage from combined CRS and HIPEC therapy.
